# A Reduction in a Drug Account

**Published:** 1915-07-17

**Authors:** 


					A Reduction in a Drug Account.
To the Editor of The Hospital.
Sir,?I am sure that many hospital pharmacists
and dispensers, who were interested in your note
of " A Eeduction in a Drug Account," would, like
myself, be disappointed with the letter signed
" W. J. B.," because of its lack of details; I can,
however, quite understand that probably the phar-
macist, Mr. Hurn, feels that he cannot make public
the details without breach of faith with his medical
staff.
I also have experienced a reduction in my drug
account, and that against a large increase in num-
ber of beds (for wounded soldiers) and a large
increase in out-patients arising from over 15,000
billeted troops.
The reason for [? the reduction in] my drug
account, I think, fitly answers the question in
your footnote to " W. J. B.'s" letter, "Could
not, then, the successful exertions of the dispenser
[italics mine] and the staff [medical staff under-
stood] have been employed before? " you ask, Sir.
I answer No! because the co-operation implied by
the word "and" was absent; but as soon as,
under stress of necessity, the mere " dispense1!
became pharmacist, and an integral part of'
medical staff, the thing was done.
So long as the " dispenser " is a mere autofli3';
machine, shut up in a box of bottles with ctl
venient hatches to throw his stuff out of, sel^1:
seen and never spoken to by the medical
economy will not be a striking product of his.'
partment; and by " economy " I do not mean V
drug account only, but everything that word1
plies, including efficiency. ^
My plea is that the man or woman who is ,
presiding genius of the " drug shop " in an ^
tution should be a person of knowledge and ^
experience, such as commands the respect of'
brethren in the craft and thus the confidence 3
esteem of his medical staff. ^
Whatever may be the economic ambition of J
pharmacist, he must supply that which is ord ^
by the staff, and he can only bring about
economies in the measure in which he can influ
the formation of those orders.
To sum up, the reduction in my drug .
has not been due to the " making of concocti^
(I like that word), but largely to the extent of
experience, which commenced before the intr? 'y
tion of the " German " drugs, and mainly ,
willing co-operation of the medical staff with \
Hospital Pharmacy
[We are glad to publish this letter, which y
admirably the point of view of the keen ^?Su
pharmacist. A proposal on the matter is fl18
our Notes this week.? Ed. The Hospital-]

				

